# Nonlinear phenomena in vertebrate vocalizations: mechanisms and communicative functions

**DOI:** 10.1098/rstb.2024.0002

**Published:** 2025-04-03

**Authors:** Mathilde Massenet, Nicolas Mathevon, Andrey Anikin, Elodie F. Briefer, W. Tecumseh Fitch, David Reby

**Affiliations:** ^1^ ENES Bioacoustics Research Laboratory, CRNL, CNRS, Inserm, University of Saint-Etienne, 42100 Saint-Etienne, France; ^2^ Division of Cognitive Science, Lund University, 223 62 Lund, Sweden; ^3^ Ecole Pratique des Hautes Etudes, University Paris-Sciences-Lettres, 75014 Paris, France; ^4^ Institut universitaire de France, 75231 Paris, France; ^5^ Behavioural Ecology Group, Section for Ecology and Evolution, Department of Biology, University of Copenhagen, DK-2100 Copenhagen Ø, Denmark; ^6^ Department of Behavioral and Cognitive Biology, University of Vienna, 1030 Vienna, Austria

**Keywords:** nonlinear phenomena, vertebrate, production, perception, evolution, vocal communication

## Abstract

Nonlinear phenomena (NLP) are acoustic irregularities that are widespread in animal and human vocal repertoires, as well as in music. These phenomena have recently attracted considerable interest but, surprisingly, have never been the subject of a comprehensive review. NLP result from irregular sound production, contribute to perceptual harshness, and have long been considered nonadaptive vocal features or by-products of sound production characterizing pathological voices. This view is beginning to change: NLP are increasingly documented in nonverbal vocalizations of healthy humans, and an impressive variety of acoustic irregularities are found in the vocalizations of nonhuman vertebrates. Indeed, evidence is accumulating that NLP have evolved to serve specific functions such as attracting listeners’ attention, signalling high arousal, or communicating aggression, size, dominance, distress and/or pain. This special issue presents a selection of theoretical and empirical studies showcasing novel concepts and analysis tools to address the following key questions: How are NLP in vertebrate vocalizations defined and classified? What are their biomechanical origins? What are their communicative functions? How and why did they evolve? We also discuss the broader significance and societal implications of research on NLP for non-invasively monitoring and improving human and animal welfare.

This article is part of the theme issue ‘Nonlinear phenomena in vertebrate vocalizations: mechanisms and communicative functions’.

## 1. Introduction

**Figure 1.**
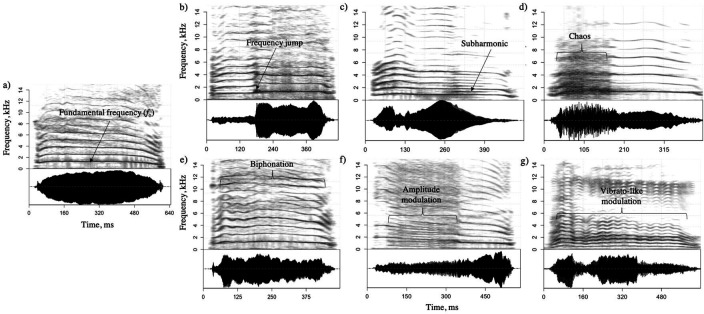
Spectrograms of domestic dog puppy whines without (*a*) and with (*b–g*) nonlinear phenomena. Frequency jumps (*b*) correspond to abrupt discontinuities in the fundamental frequency *f*_o_. Subharmonics (c) correspond to spectral bands harmonically related to *f*_o_, here occurring at one-half of *f*_o_. Deterministic chaos (*d*) appears as noisy sections where *f*_o_ is difficult to track. Biphonation (*e*), as in horse whinnies, involves a laryngeal and a supra-laryngeal sound source vibrating at two different frequencies, *f*_o_ and *g*_o_, and induces visible sidebands at *n×g*_o_ ± *m×f*_o_ (where *f*_o_ is lower than *g*_o_, *m* and *n* are integers). Similarly, amplitude modulation (*f*), as seen in, e.g., dog growls, induces sidebands visible at *n×f*_o_ ± *m*×*j*_o_ (where *f*_o_ is higher than *j*_o_). Finally, vibrato-like modulation corresponds to frequency (and amplitude) modulations, as in, e.g., goat bleats. Spectrograms were generated with the *spectrogram*() function from *soundgen* R package [3].

Why are puppies’ whines and human babies’ cries so hard to ignore? Why are screams in horror movies so terrifying? Why is a lion’s roar so intimidating, and why does heavy metal singing sound so aggressive? A common answer to these questions may lie in an acoustic feature that all these sounds share: they contain perceptually harsh vocal elements [1], called *nonlinear phenomena* (hereafter abbreviated ‘NLP’) [2] (see figure 1 for details on the different types of NLP).

These acoustic phenomena result from relatively unstable oscillations of vibrating systems, such as the syrinx of birds, the larynx of mammals or the vibrating membrane of a guitar amplifier, which are often caused by slight changes in the configuration of these systems or by overdriving them beyond the limits of stable vibration [4–6]. As a result, NLP in human vocal signals have historically been seen as nonadaptive by-products of human pathological phonation (e.g. diplophonia [7]), or as a consequence of lack of vocal control in classical singing [8].

However, over the past 20 years, this restrictive view of NLP in vocal communication has been increasingly challenged. Indeed, the fact that NLP also frequently occur in the vocalizations of healthy vertebrates, including birds, amphibians and mammals, and are characteristic of certain music styles, such as hard rock music, suggests that the production of NLP may serve important biological communicative functions. Several hypotheses have been proposed to explain these adaptive functions [9], most of which remain untested. These hypotheses include attracting the attention of listeners and preventing habituation to harsh vocal signals [10–13]. NLP have also been suggested to signal high arousal in contexts associated with aggression, pleasure, distress or pain [1,14–17] and to communicate information about the size and dominance of vocalizers [1,18].

Despite a growing research interest in NLP among multiple research disciplines (e.g. physical voice sciences [19], medical sciences [20], behavioural sciences [18], social sciences [21] and evolutionary biology [22]), our understanding of the mechanisms of production, communicative functions and evolution of NLP remains rather limited. Until recently, the field has been hindered by a lack of an over-arching theoretical and experimental framework. Specifically, researchers have lacked agreed-upon definitions of NLP, used oversimplified biomechanical models that lacked realistic grounding in functional anatomy for the simulation of NLP production, and employed unsatisfactory methods for NLP visualization and analysis. In addition, most playback studies investigating the communicative functions of NLP have been limited to presenting animals with natural calls [10,23] and have thus been unable to disentangle the effects of NLP on animals’ responses from those caused by other covarying acoustic features such as call frequency [24]. Excitingly, recently emerging tools now help researchers to visualize, identify and quantify NLP in natural vocalizations and to investigate their biomechanical origins in vocal production using more anatomically informed models. Furthermore, recent ground-breaking parametric sound synthesis algorithms enable us to systematically manipulate these vocal phenomena and to directly test their signalling function in human and animal communication by examining their effects on the behavioural or physiological responses of listeners [3].

The production, communicative functions and evolution of NLP in vertebrate vocalizations undoubtedly remain emerging ‘hot’ topics, as established during a recent 3-day conference on NLP held in Saint-Etienne, France in June 2023, where international experts in the field agreed on the need to take stock of recent advances through a comprehensive review, to assess the state-of-the-art situation, and to open pathways for future work. The outcome of this collaborative effort is this special issue, which fills a large existing gap by providing a comprehensive and multilevel overview of past and ongoing research on NLP in a wide variety of species, including humans. It comprises 22 articles authored by a group of international specialists that directly address one or more of the following areas:

1. Definitions, history and methodologies for studying NLP.
2. Production mechanisms underlying NLP.
3. NLP as a cue to pathology.
4. NLP as adaptive features of vertebrate vocal communication.
5. Ontogeny of NLP.

### (a) Definitions, history and methodologies for studying nonlinear phenomena

The special issue begins with a historical perspective on research on NLP, authored by W. Tecumseh Fitch [25]. Based on interviews with key figures in the field and his own experience, Fitch explains how the concepts and mathematical principles of nonlinear dynamics—already widely applied in disciplines such as physics, chemistry, physiology and ecology—were introduced to human voice sciences, and then rapidly extended to vocal communication among other vertebrates. In the second article of this issue, Muir *et al*. [5] document how this research field has gained increasing attention from scientists over the past 20 years. They identify more than 200 articles on NLP in mammalian vocal communication, but note that these studies remain highly biased towards a small number of taxonomic orders (i.e. artiodactyls, carnivores and rodents).

NLP in vertebrate vocal signals consist of several main types: frequency jumps, subharmonics, biphonation, amplitude modulation, frequency modulation (or vibrato) and deterministic chaos (figure 1). This terminology corresponds to modern terms commonly used by researchers, particularly bioacousticians, since the early 2000s [5]. In their review, del Olmo *et al*. [26] also provide detailed definitions of NLP from the perspective of physics. They explain how the principles of oscillator theory and nonlinear dynamics offer a fundamental framework for understanding the transitions or ‘bifurcations’ (i.e. Hopf, period-doubling and secondary Hopf bifurcations) between specific patterns of oscillation (‘attractors’) and discuss how these apply to different types of NLP in vocal signals. Svec & Zhang [27] further elaborate on how this framework can be directly applied to human voice sciences, including acoustically characterizing pathological, dysphonic voice (see details in §1c) or exploring diversity among singing registers. To visually represent bifurcations of coupled oscillators that lead to NLP occurrence in acoustic signals, del Olmo *et al.* [26] also provide a comprehensive explanation of Arnold tongue diagrams, highlighting how they serve as a useful tool to map the observed attractors on a plane defined by frequency ratio and coupling strength between two oscillators.

Anikin & Herbst [28] then present a comprehensive review of the available methods for identifying and quantifying NLP in both human voice and bioacoustics. Specifically, they discuss best practices and limitations of manual NLP annotation, review the relevant literature on the analysis and synthesis of NLP, and suggest practical recommendations and guidelines for future work. Crucially, this review encourages a broader integration of techniques across disciplines, including human voice sciences and bioacoustics, to make future research on NLP as effective and replicable as possible.

### (b) Production mechanisms underlying nonlinear phenomena

We now turn to the biological mechanisms underlying the production of NLP, which are essential for grounding the physical concepts outlined in §1a in their biological substrates, namely, vocal production systems. We start with birds, in which the vocal organ, called the syrinx, exhibits considerable anatomical diversity, with up to three pairs of functional vocal folds, each acting as independent sound sources, and eight pairs of muscles controlling them (in songbirds). In this issue, Amador *et al.* [4] show how this complexity supports a wide range of NLP, providing unique opportunities to explore rich nonlinear dynamics in vocal production. The review by Muir *et al.* [5] then discusses NLP production in other nonhuman vertebrates. This typically involves vibrating vocal folds under relatively high subglottal pressures, asymmetries in the vocal fold configuration and/or interactions between the larynx and resonant cavities (i.e. the supralaryngeal vocal tract, including both nasal and oral cavities). Crucially, the authors point out that existing research is strongly biased towards human vocal production, with only a dozen studies reported on nonhuman mammals across just a few clades. They highlight that the methods commonly used for studying NLP production *in vivo*, such as laryngoscopy, are challenging to apply to nonhuman animals, and encourage future studies to address this gap in order to develop a broader comparative framework.

While the first two reviews of this section provide broad overviews of the mechanisms underlying the production of NLP in vertebrate vocalizations, subsequent articles focus more specifically on the production of certain types. These include discussions on transitions between different vocal registers, which often result in abrupt fundamental frequency jumps. More specifically, Herbst & Elemans [29] review the biophysical underpinnings of vocal registers and the signatures they leave in vocal fold kinematics and acoustics in humans. They show that vocal registers are not unique to the human voice and suggest that such registers may represent a fundamental trait of vocal production in other clades. Moreover, they argue that vocal registers may have been favoured by selection to extend and diversify the acoustic signalling space of vertebrates.

This idea is further illustrated in the following article, where Herbst *et al.* [30] provide deeper insights into how rapid frequency jumps (akin to human yodelling) occur in the vocalizations of New World monkeys. Using a combination of acoustic and electroglottographic (EGG) recordings *in vivo*, excised larynx investigations of vocal fold dynamics, and computational modelling, the authors identify two key mechanisms involved in the production of frequency jumps. The first mechanism, involving only vocal fold vibration, produces low frequencies and is analogous to that observed in human yodelling. In contrast, the second mechanism, which involves vocal membranes—structures absent in humans—produces much higher-frequency oscillations.

Finally, the last article of this section focuses on biphonation, a remarkable type of NLP involving the simultaneous production of two distinct, non-harmonically related fundamental frequencies, which is commonly observed in the calls of phylogenetically distant animals ranging from amphibians to whales. Lefèvre *et al*. [31] review the current state of knowledge on biphonation, exploring its biomechanical origins—including asymmetrical vocal fold oscillations, aerodynamic whistles, involvement of secondary structures (vocal membranes in nonhuman primates, or bilateral specializations in some birds)—as well as its potential signalling functions, as detailed in §1d.

### (c) Nonlinear phenomena as a cue to pathology

The above overview of the biomechanical origins of NLP indicates that they typically result from ‘irregular’ phonation. Accordingly, NLP have long been regarded as mere side effects/nonadaptive features of animal vocal repertoires characterizing pathological states in vocalizers. This is illustrated in the next review, authored by Svec & Zhang [27], who describe how anatomical abnormalities in the vocal apparatus typically cause human voice pathologies characterized by the presence of NLP. They highlight how the theory of nonlinear dynamics can serve as a powerful framework for the clinical diagnosis and the treatment of such voice disorders. Here again, descriptions of the clinical causes of pathologies underlying NLP production are strongly biased towards humans.

Indeed, studies on the potential use of NLP as a cue to pathology in nonhuman animals are surprisingly rare, despite the broad applications such work would potentially offer to animal conservation and/or husbandry. This special issue, however, showcases two examples highlighting the link between animal health and the production of NLP. The first is an empirical study conducted by Morandi *et al.* [32] in an endangered species, the African penguin (*Spheniscus demersus*). They show that the occurrence of NLP in chick begging calls increases when individuals are affected by respiratory infections, particularly bacterial infections and aspergillosis. The second is reported in Daniel Blumstein’s review [33] of his extensive research on the behavioural ecology of yellow-bellied marmots (*Marmota flaviventer*): animals infected with intestinal parasites produce alarm calls characterized by higher levels of NLP compared with those produced by nonaffected individuals. These papers suggest a rich potential for further investigations of the relationship between NLP and health, and therefore welfare, in nonhuman animals.

### (d) Nonlinear phenomena as adaptive features of vertebrate vocal communication

In this section, we explore the idea that, rather than just being ‘unwanted’ or involuntary by-products of irregular phonation, NLP can convey significant biological information. Asymmetries in the vocal system, which often yield NLP, can be permanent, but they can also be temporary and reflect transient physiological states. For example, highly aroused animals (e.g. facing predation risk and/or seeking assistance) are likely to vocalize with high subglottal pressure and/or strong tension in vocal muscles. This configuration of the vocal apparatus often destabilizes its otherwise relatively stable vibratory pattern, ultimately leading to the production of NLP. As a consequence, it has been argued that NLP may serve as cues to high arousal, particularly in negatively valenced contexts such as aggression [1], distress [18] or pain [34,35]. This hypothesis is extensively discussed in Blumstein’s review [33], already mentioned above, where he highlights the role of NLP for signalling fear to conspecifics in marmots: as they emerge from their natal burrow, pups can emit ‘fear’ screams typically containing NLP that are particularly evocative to other marmots.

The special issue then continues with a set of empirical studies that further illustrate the adaptive value of NLP as cues to, or signals of, the emotional and/or motivational state of vocalizers. First, Massenet *et al.* [14] demonstrate that the production of NLP in the whines of domestic dog puppies increases with the time since their separation from their mother. Then, Corvin *et al*. [15] show that human babies’ cries emitted in a context associated with acute pain, such as vaccination, are characterized by higher levels of NLP than those emitted in contexts associated with mere discomfort, such as bathing. They also use state-of-the-art methods of parametric sound synthesis (the R package *soundgen* [3]) to prepare highly realistic-sounding synthetic baby cries with controlled levels of NLP, in order to test the specific effect of NLP on listeners’ pain perception. Psychoacoustic experiments reveal that the presence of NLP, and especially the presence of deterministic chaos, affect the level of pain that listeners perceive the crying baby to be experiencing.

The strong and highly specific perceptual effects of NLP are further corroborated by the evidence presented by Valente *et al.* [16], who use a comparable experimental approach to investigate the extent to which NLP convey pain information in human childbirth vocalizations. This study points to a more general role of NLP as cues to pain in human nonverbal vocalizations. Finally, Fournier *et al.* [17] explore the occurrence of NLP in the vocal repertoires of humans’ closest relatives: bonobos and chimpanzees. Nonlinearities are more frequent in the call types given in negatively valenced contexts (i.e. aggression) than in those emitted in positive or neutral contexts (feeding and food anticipation, respectively), as previously described in human vocalizations [36]. The authors also report that, in bonobos only, the presence of NLP in calls increases with arousal during agonistic interactions.

While this set of studies supports the idea that NLP convey information about the vocalizer’s emotional or motivational state, other non-mutually exclusive signalling functions of NLP have been suggested to explain their adaptive value in animals’ vocal communication systems. Muir *et al*. [5] provide a comprehensive overview of these possible communicative functions, largely focusing on the vocalizations of nonhuman mammals. They suggest four other potential functions: communicating the vocalizer’s identity, expressing social–sexual traits (e.g. physical condition, fertility or social rank), gaining attention, and reducing habituation in listeners. Finally, Lefèvre *et al*. [31] review the potential communicative functions of biphonation. Importantly, they discuss how biphonation may enable animals to communicate multiple messages simultaneously, by enhancing signal complexity and individual signatures and providing directional movement cues.

### (e) Ontogeny of nonlinear phenomena

In this section of the special issue, we discuss the extent to which the occurrence of NLP varies throughout animals’ lifespans. Muir *et al.* [5] point out that past research investigating NLP ontogeny has been strongly biased towards a small number of mammalian clades, i.e. cetaceans, rodents and primates including humans. The special issue brings new exciting results in domestic dogs [14], indris [37] and elephant seals [38]. While all of these studies document an overall decrease in the production of NLP during development, they also found that the specific ontogenetic pathway of each type of NLP may be different. In other words, some NLP types may decrease with age, whereas others may increase or remain stable. For example, Massenet *et al*. [14] report that chaos in puppy whines strongly decreases in the course of development, while biphonation increases. De Gregorio *et al*. [37] show that the production of all NLP types in indri calls decreases with age, except for subharmonics, which remain stable. Finally, Linossier *et al*. [38] also show that the occurrence of vibrato in elephant seal pup contact calls decreases as these individuals grow, whereas biphonation, chaos and subharmonics become more common. These new findings support the general contention that better vocal control in adults leads to lower NLP occurrence, albeit with selective stabilizations or increases in particular NLP types that appear likely to serve specific communicative functions (as discussed in §1d).

## 2. Towards a theory of the evolution of nonlinear phenomena occurrence and function in animal vocal communication?

**Figure 2.**
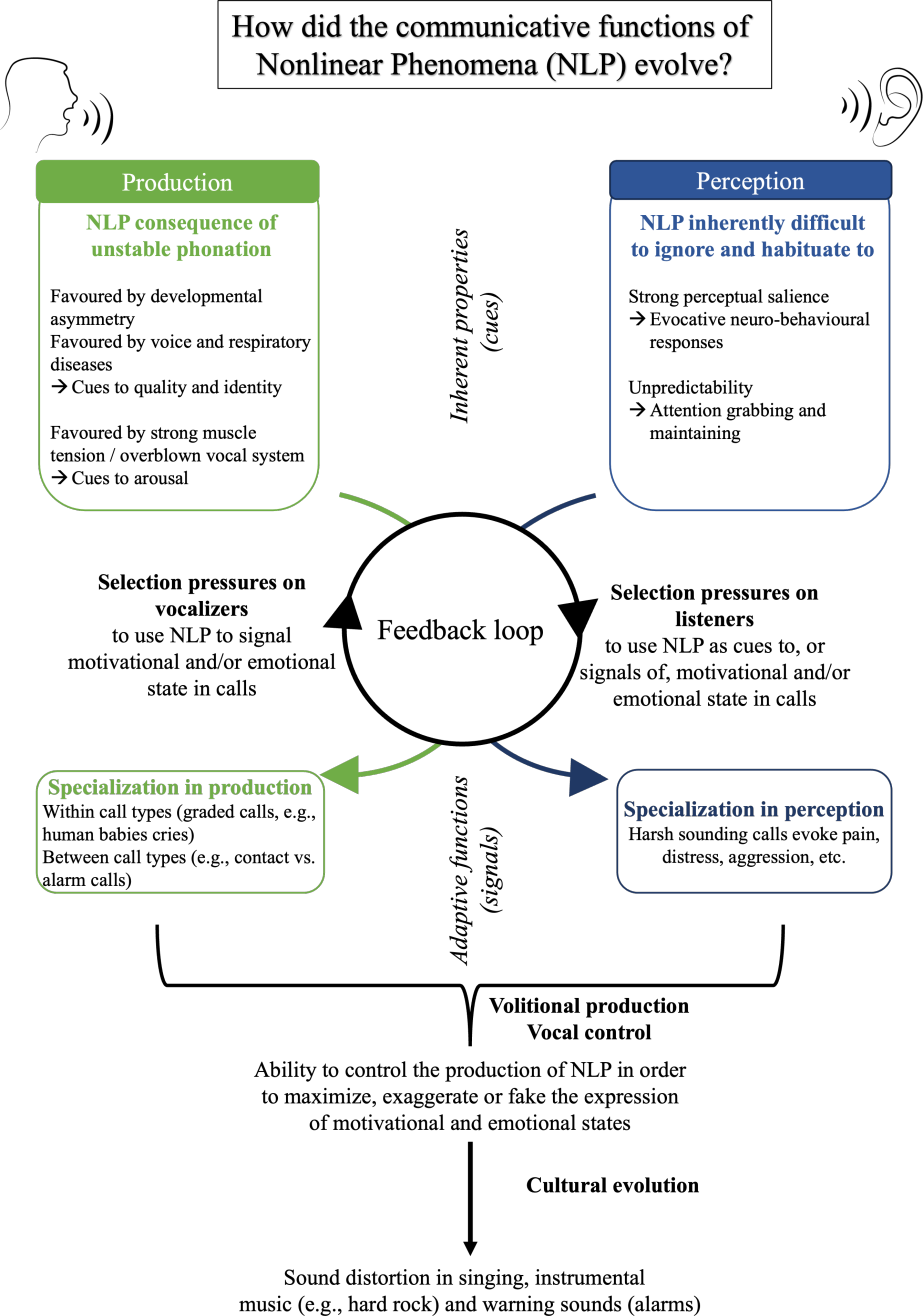
Possible scenarios underlying the evolution of nonlinear phenomena (NLP) as functional cues in vertebrate vocal repertoires.

The comprehensive methodological, theoretical and phylogenetic diversity brought together in this special issue allows us to speculate as to how NLP may have evolved in the vocal repertoires of vertebrates (figure 2). We suggest that the adaptive roles of NLP can be seen as exaptations [39] of pre-existing properties of vocal and auditory mechanisms—at production (i.e. overdriving vocal systems, asymmetries) and perception (i.e. acoustic salience of NLP) levels.

At the production level, NLP are the consequence of instabilities occurring in a complex vocal system composed of elements (vibrating membranes, muscles, cartilages, and the air in the vocal tract) that act as independent or interconnected oscillators (as discussed in §§1a,b). Instabilities can be permanent as a result of abnormalities in the vocal organ, or transient owing to physiological variation in the emotional state of the vocalizer, potentially involving momentary loss of vocal control. In these contexts, NLP are thus likely to constitute a *cue* to pathology or high arousal, even in the absence of any anatomical specializations for their production. As a consequence, selective pressures are expected to act on both vocalizers and receivers, turning the presence of NLP into a biological *signal*. More specifically, cues inherently associated with the presence of NLP may have favoured their specialization as a communicative signal in the communication system of some vertebrates via ritualization, as further discussed by Rendall in this issue [40]. These pressures may lead to the graded occurrence of NLP within calls, as reported in domestic dog puppies [14] and human baby cries [41], or to their systematic presence in specific calls types, such as alarm calls in marmots [33] or harsh roars in red deer [42]. However, Rendall also contrasts this idea of *functional production* of NLP to that of a *functional avoidance* [40]. For example, the production of NLP may reflect relatively low quality in males during male competition and female access [43], which should favour males that suppress these cues.

In line with this idea of functional avoidance of NLP, a recent study has suggested that anatomical simplification of the human vocal apparatus—i.e. the loss of vocal membranes and air sacs—evolved to favour the emergence of stable tonal speech and facilitate its intelligibility to listeners [22]. However, Anikin *et al*. in this issue [44] demonstrate that the presence of NLP in isolated vowels and even entire sentences does not necessarily affect speech intelligibility as long as normal voicing distinctions and intonation are preserved. They suggest that evolutionary pressures to eliminate NLP from the human vocal repertoire as speech evolved might have been limited, and that the main evolutionary change was to bring these complex acoustic phenomena under better vocal control.

At a perceptual level, NLP are inherently unpredictable and rough [1,9], potentially making them well suited for attention-grabbing functions, over and above the fact that they signal high arousal and thus potentially important interactions or events. Arnal & Gonçalves, in this issue [45], review converging evidence from human and nonhuman animals indicating that the perceptual properties of NLP, especially their ability to evoke the sensation of auditory roughness, are ideally adapted to induce efficient sensory, emotional and behavioural responses. Evolutionary pressures to use NLP as functional cues to caller attributes or states may have strengthened these perceptual associations in listeners, who now typically associate vocal harshness with aggression [1], distress [18] or pain [15,16]. In other words, selective pressures on production and perception, likely entrained in a positive feedback loop, could have acted both on vocalizers to express high emotional and/or motivational states, and on listeners to detect such information in calls and respond adaptively.

In species with advanced vocal control abilities, such as humans, volitional NLP production (bottom section of figure 2) clearly plays a role in exaggerating or faking the expression of vocalizers’ motivations and emotions in order to manipulate listeners’ reaction and subsequent behaviour [34,46,47]. This is typically seen in acting or music: actors often emit screams containing relatively high levels of NLP to express simulated pain [34], and the presence of NLP in music typically evokes intense emotions in listeners [21]. In this issue, Bryant & Smaldino [6] highlight how sound distortion leading to the production of NLP has arisen culturally in specific music styles such as hard rock music, which is often perceived as aggressive. These studies show that the role of NLP in sound communication, at least in humans, has continued to evolve culturally.

## 3. Conclusions and future directions

In this compilation of reviews, perspectives and empirical articles, we take stock of past and current research on a topic that has recently reached a state of near-maturity. This issue provides a unique opportunity to clarify definitions in NLP, highlight major methodological advances, report experimental studies investigating how NLP function in vertebrate calls and discuss their evolution in communication systems. It also allows us to identify important gaps in current knowledge. As pointed out by Muir *et al*. [5], there is an urgent need to broaden both observational and experimental studies to more diverse taxonomic groups in order to provide a robust comparative framework for future phylogenetic investigations at both production and perception levels. Such investigations would allow researchers to address important open questions from a broader comparative perspective: when during evolution did NLP appear within calls or within specific call types? Can we identify specific selection pressures that tend to drive the emergence and evolution of NLP? To what extent are vocalizers able to control NLP, and thus exaggerate or fake the communication of bio-social attributes, emotions or motivations? Other than in humans, in which other clades and why did such abilities evolve?

To conclude, we believe that this collection of articles offers a robust theoretical and methodological framework that we hope will encourage future research in a wide range of disciplines, including physics, voice sciences, bioacoustics and, more generally, evolutionary biology.

## Data Availability

This article has no additional data.
